# Allotrope-dependent physicochemical and optical properties of red and grey selenium nanoparticles

**DOI:** 10.1039/d6ra01409g

**Published:** 2026-04-20

**Authors:** Aya Ferroudj, Attila Csík, József Prokisch

**Affiliations:** a Nanofood Laboratory, Department of Animal Husbandry, Institute of Animal Science, Biotechnology and Nature Conservation, Faculty of Agricultural and Food Sciences and Environmental Management, University of Debrecen 138 Böszörményi Street Debrecen 4032 Hungary ferroudj.aya@agr.unideb.hu; b Doctoral School of Animal Husbandry, University of Debrecen Böszörményi Street 138 Debrecen 4032 Hungary; c HUN-REN Institute for Nuclear Research Bem Tér 18/c Debrecen 4026 Hungary

## Abstract

Selenium nanoparticles (SeNPs) are increasingly being investigated as safer and more bioavailable alternatives to conventional selenium compounds; however, the influence of selenium allotropy on their physicochemical and optical properties remains poorly understood. In this study, red and grey SeNPs were synthesized and comparatively characterized using scanning electron microscopy (SEM), energy-dispersive X-ray spectroscopy (EDS), X-ray diffraction (XRD), Raman spectroscopy, and fluorescence spectroscopy. Red SeNPs exhibited predominantly spherical morphology with an average diameter of 218 ± 24 nm and an amorphous structure, whereas grey SeNPs formed needle-like nanostructures with a mean length of 575 ± 202 nm and a trigonal crystalline structure. Raman spectra showed a broad band at 250–255 cm^−1^ for red SeNPs and a sharp peak at 233 cm^−1^ for grey SeNPs, corresponding to amorphous and trigonal selenium, respectively. Grey SeNPs displayed significantly stronger fluorescence emission at 430–450 nm than red SeNPs, indicating that increased crystallinity and structural ordering enhance fluorescence activity. These findings demonstrate that selenium allotropy governs morphology, crystallinity, and photophysical behavior and provide a physicochemical basis for understanding the different functional performances of selenium nanomaterials.

## Abstract 2

Selenium nanoparticles (SeNPs) are promising alternatives to conventional selenium compounds; however, the influence of selenium allotropy on their physicochemical and optical properties remains poorly understood.

## Graphical abstract

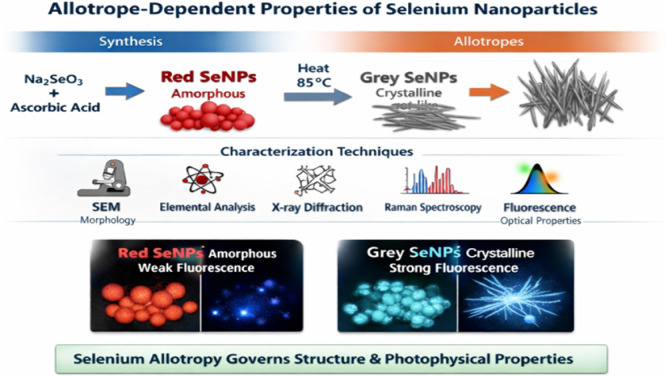

## Introduction

Selenium (Se) has found extensive applications in diverse fields, ranging from electronics, glass production, pigments, and agriculture, to nutrition and pharmaceuticals.^1–4^ Chemically, selenium can exist in several oxidation states, including Se^(−II)^, Se^(+IV)^, and Se^(+VI)^, which makes it a highly versatile element in both industrial and biological systems.^5^ In living organisms, Se is an essential micronutrient incorporated in the form of selenocysteine (SeCys) and selenomethionine (SeMet); these amino acids contribute to the catalytic sites of selenoproteins that play critical roles in antioxidant defence, redox regulation, and immune function.^6^ However, selenium is characterized by a narrow margin between essentiality and toxicity: while deficiency is linked to disorders such as cardiomyopathy, immune dysfunction, and reproductive failure, excessive intake can cause selenosis and oxidative damage.^7–9^ This duality has motivated the search for safer, more bioavailable forms of selenium suitable for dietary and biomedical applications.

Selenium nanoparticles (SeNPs) have emerged as promising alternatives to traditional selenium compounds. Compared with inorganic salts such as sodium selenite or selenate, SeNPs are reported to exhibit lower toxicity, improved bioavailability, and higher biological activity.^10^ SeNPs have been widely studied for their antioxidant, anticancer, antimicrobial, and anti-inflammatory properties, as well as for applications in drug delivery and imaging.^11–13^ A unique characteristic of SeNPs is their ability to exist in different allotropes, with amorphous red and crystalline grey forms being the most common.^14,15^ Red selenium is typically amorphous and less stable, whereas grey selenium adopts a crystalline structure and represents the thermodynamically stable phase.^16^ These allotropes differ not only in atomic arrangement but also in morphology, surface properties, electronic structure, and optical behavior, making their characterization essential for both biological and technological applications, since the allotropic phase can directly influence their physicochemical reactivity and biological performance.

To understand these differences, a comprehensive physicochemical characterization is required. Despite growing interest in selenium nanomaterials, many previous studies have focused primarily on their biological activity or employed limited physiochemical characterization, often restricted to morphology (SEM), elemental composition (EDS), or crystallinity (XRD).^17–20^ Scanning electron microscopy (SEM) and energy-dispersive X-ray spectroscopy (EDS) provide essential information on particle morphology and elemental composition, while X-ray diffraction (XRD) enables definitive identification of amorphous *versus* crystalline phases.^21,22^ At the molecular and electronic levels, Raman spectroscopy offers sensitive discrimination between selenium allotropes through their characteristic vibrational modes, allowing the identification of amorphous and crystalline phases of selenium.^23^ Amorphous red selenium typically displays broad Raman bands in the 250–255 cm^−1^ region, while grey crystalline selenium with both types; trigonal rings and monoclinic are characterized by a sharp bands near to 233–251 cm^−1^ respectively.^24,25^ Fluorescence or photoluminescence (PL) spectroscopy provides insight into the electronic states, defect levels, and surface-related emissive centers in nanostructures.^21^ Selenium nanostructures have been reported to display photoluminescence in the visible range, with emission features strongly dependent on particle size, allotropy, and quantum confinement effects.^26^ Interestingly, while amorphous SeNPs are often associated with strong fluorescence, recent studies indicate that crystalline selenium nanostructures can also exhibit significant optical activity, challenging the conventional view that crystallinity quenches emission.^24^

Although several studies have reported the synthesis and characterization of selenium nanoparticles, the majority have focused either on their biological activity or on simple UV-vis characterization.^27^ Direct comparative studies integrating physicochemical and optical features of red and grey SeNPs remain limited, even though these complementary techniques are crucial for understanding their phase-dependent optical responses. Moreover, it is not yet fully understood whether crystallinity enhances or suppresses fluorescence in selenium nanostructures, since both amorphous and crystalline forms have been reported to display photoluminescence under certain conditions.^23^ Therefore, the present study aims to comprehensively investigate the structural, morphological, and optical properties of red (amorphous) and grey (crystalline) selenium nanoparticles. SEM and EDS were employed to examine particle morphology and elemental composition, while XRD and Raman spectroscopy were used to confirm their allotropic structure. Fluorescence spectroscopy was applied to evaluate their excitation-dependent emission behavior and concentration-dependent optical response. By integrating these complementary analytical techniques, this work seeks to elucidate how selenium allotropy governs the physicochemical and optical properties of SeNPs and to provide insights relevant to both fundamental materials science and potential biomedical and optical applications.

## Methods and materials

### Preparation of selenium nanoparticles

**Fig. 1:**
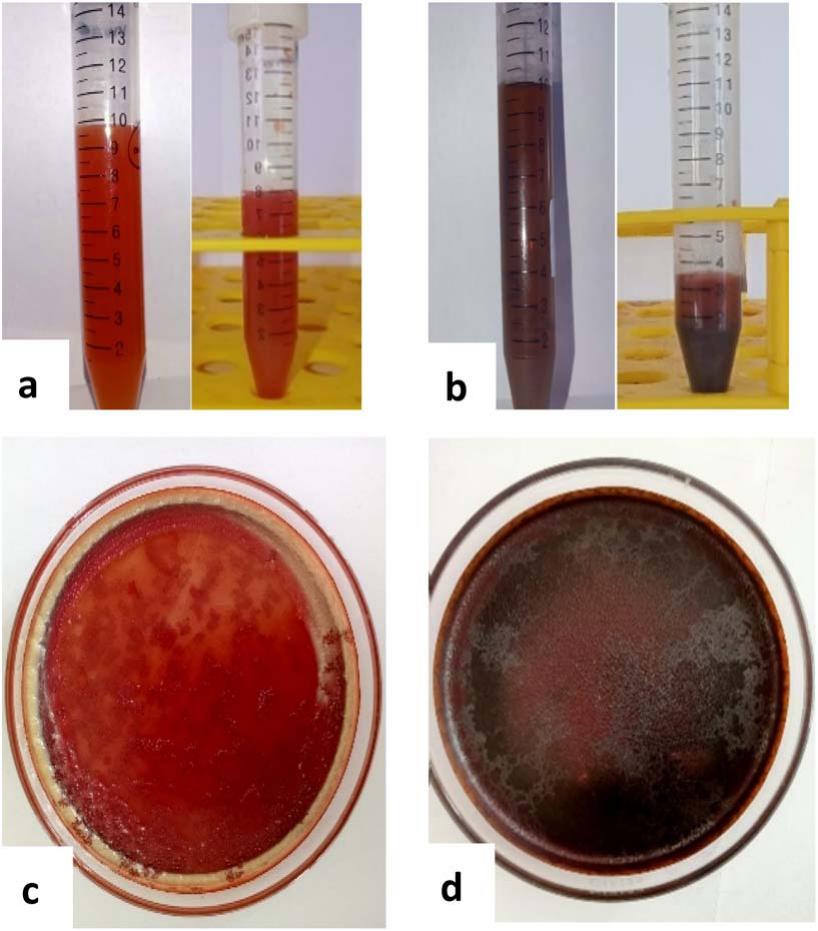
Visual appearance of selenium nanoparticles in two allotropes. (a) Red SeNP suspension, (b) grey SeNP suspension, (c) red SeNP solid after freeze-drying, and (d) grey SeNP solid after freeze-drying. Suspensions were used for fluorescence measurements, while solid forms were analysed by SEM-EDS; XRD and Raman spectroscopy.

Selenium nanoparticles (SeNPs) were synthesized in aqueous solution by chemical reduction of sodium selenite using ascorbic acid. Sodium selenite was dissolved in deionized water to prepare stock solutions with selenium concentrations of 1000, 700, and 500 mg L^−1^. Reduction was initiated by the gradual addition of a 1% (w/v) ascorbic acid solution under continuous magnetic stirring at room temperature. The formation of red selenium nanoparticles was confirmed visually by the appearance of a characteristic red coloration. For analyses requiring solid material, red SeNP suspensions were purified by centrifugation at 6000 rpm for 10 min, repeated three times, with washing using distilled water after each centrifugation step to remove residual reactants. The purified nanoparticles were subsequently freeze-dried to obtain dry red SeNP powder. Grey selenium nanoparticles were obtained by thermal transformation of red SeNPs in aqueous suspension. Red SeNP suspensions were heated at 85 °C for 10 min, following the reported protocol,^28^ resulting in a visible color change indicative of conversion to the grey selenium allotrope. The formation of the two selenium allotropes is governed by different nucleation and growth mechanisms. The rapid reduction of selenite by ascorbic acid at room temperature kinetically favors the formation of metastable amorphous red selenium. Under these conditions, rapid nucleation and limited atomic mobility prevent the rearrangement of selenium atoms into an ordered lattice, resulting in isotropic growth and predominantly spherical particles. In contrast, heating the red SeNP suspension at 85 °C promotes atomic diffusion and structural reorganization into the thermodynamically stable trigonal grey selenium phase. This phase consists of helical Se–Se chains that favor anisotropic crystal growth, producing elongated needle-like nanostructures. Both liquid suspensions and freeze-dried powders were used for subsequent analyses, depending on the requirements of each technique Fig. 1.

### Scanning electron microscopy (SEM) and energy-dispersive X-ray spectroscopy (EDS)

The morphology and surface structure of red and grey selenium nanoparticles were examined using scanning electron microscopy (SEM). Measurements were performed at the Institute for Nuclear Research (ATOMKI) using a dual-beam focused ion beam-scanning electron microscope (FIB-SEM, Thermo Fisher Scientific, Model: Scios 2, Waltham, MA, USA). Freeze-dried SeNP powders (1000 mg L^−1^) were mounted on carbon adhesive tape prior to imaging. Particle size analysis was conducted by measuring particle dimensions directly from SEM micrographs using image analysis software. Elemental composition was analyzed using an energy-dispersive X-ray spectroscopy (EDS) system (Bruker Quantax) integrated with the SEM. EDS spectra were collected to confirm the elemental composition of the selenium nanoparticles and to assess the presence of potential impurities.

### X-ray diffraction (XRD) analysis

The crystalline structure of red and grey selenium nanoparticles was investigated by X-ray diffraction (XRD) using a Rigaku SmartLab diffractometer equipped with Cu Kα radiation (*λ* = 0.154 nm). Measurements were carried out in *θ*–2*θ* scanning geometry, with the X-ray tube operated at 45 kV and 200 mA. Freeze-dried SeNP samples (1000 mg L^−1^) were gently ground to a fine powder using a mortar and pestle and then pressed into the sample holder. Diffraction patterns were recorded over a 2*θ* range of 15–75° to identify the structural phase and degree of crystallinity of the samples. Phase identification was performed by comparison with standard reference patterns from the ICDD database.

### Raman spectroscopy

Raman spectroscopy was used to characterize the vibrational properties and allotrope-dependent structural features of selenium nanoparticles. Raman spectra were recorded using a LabRAM HR Evolution Confocal Raman Microscope (Horiba Ltd, Kyoto, Japan). Measurements were performed on 1000 mg L^−1^ freeze-dried red and grey SeNP powders using a 532 nm excitation laser. Spectra were collected over the relevant Raman shift range to identify characteristic selenium vibrational modes associated with amorphous and crystalline structures.

### Fluorescence spectroscopy

The optical properties of selenium nanoparticles were evaluated using fluorescence spectroscopy. Measurements were carried out on SeNP aqueous suspensions with selenium concentrations of 500, 700, and 1000 mg L^−1^. Fluorescence spectra were recorded using a Jasco FP-8500 spectrofluorometer (Jasco, Okla-homa, USA). Excitation–emission matrix (EEM) measurements were performed with excitation wavelengths scanned over 320–480 nm, and corresponding emission spectra were collected to evaluate excitation-dependent fluorescence behavior. Fluorescence intensity data were analyzed as a function of selenium concentration, and the resulting relationships were fitted using polynomial models.

## Results

The physicochemical and optical properties of red and grey selenium nanoparticles (SeNPs) were systematically investigated using a combination of scanning electron microscopy (SEM), particle size analysis, energy-dispersive X-ray spectroscopy (EDS), X-ray diffraction (XRD), Raman spectroscopy, and fluorescence spectroscopy. This multitechnique approach was employed to elucidate allotrope-dependent differences in morphology, structure, elemental composition, and optical behavior.

### SEM analysis

**Fig. 2:**
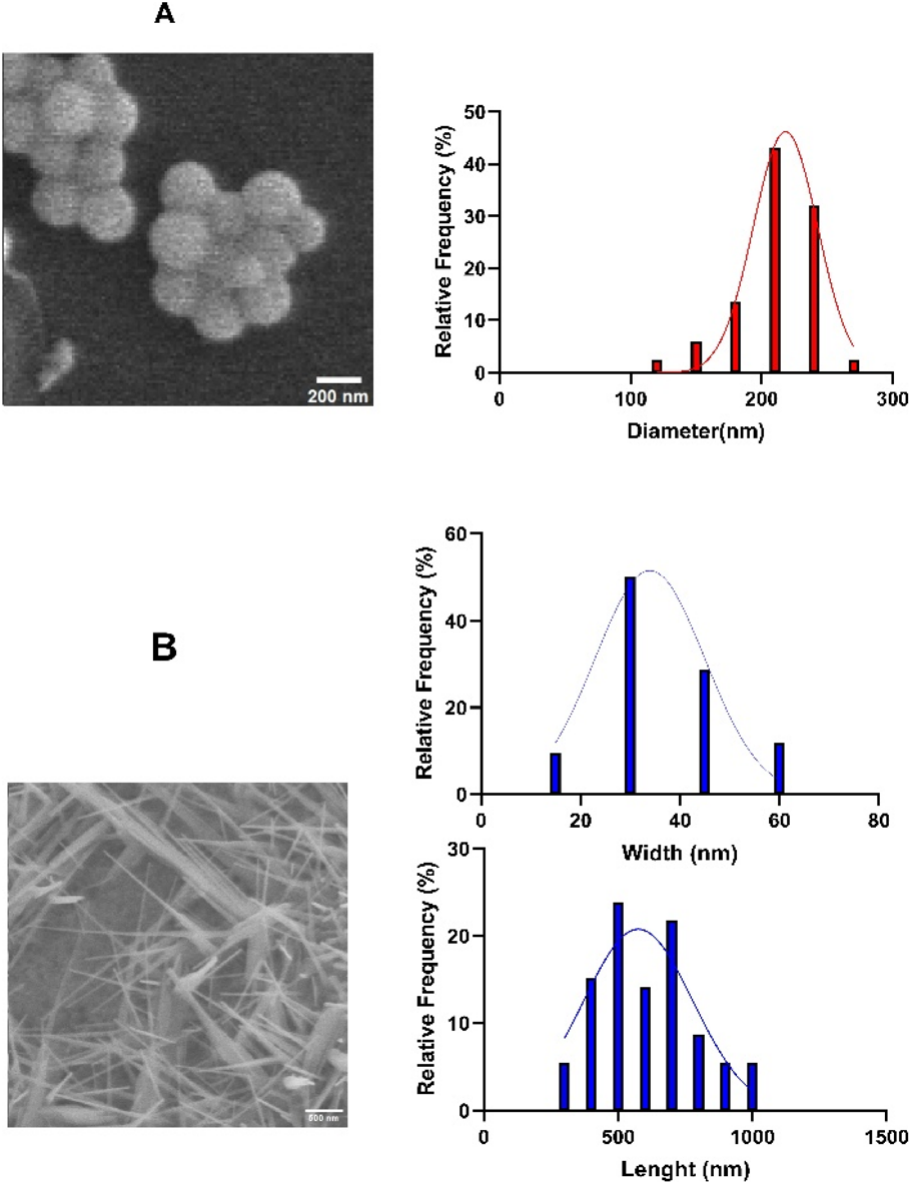
SEM images of (A) red SeNPs and diameter distribution histogram; (B) grey SeNPs and length and width distribution histograms.

To examine allotrope-dependent differences in morphology and particle dimensions, scanning electron microscopy (SEM) was employed to characterize the surface structure and size distribution of red and grey selenium nanoparticles Fig. 2.

SEM analysis revealed pronounced morphological differences between the two selenium allotropes. Red SeNPs exhibited a quasi-spherical to spherical morphology, forming compact nano-aggregates composed of smaller primary particles with smooth, non-faceted surfaces. Particle size analysis based on SEM images indicated a narrow and unimodal size distribution, with apparent particle diameters predominantly in the range of 200–220 nm, with a mean diameter 218 ± 24 nm. These dimensions represent aggregated nanoparticle domains typical of dried amorphous systems.

In contrast, grey SeNPs displayed a highly anisotropic, needle-like morphology, forming interconnected networks of elongated nanostructures. Due to their non-spherical shape, size analysis was performed by separately evaluating length and width. The nanoneedles exhibited lengths mainly between 400 and 900 nm, with a mean value 575 ± 202 nm, while widths were significantly smaller, predominantly 20–50 nm and average of 33.9 ± 11. This pronounced aspect ratio reflects directional crystal growth, which is characteristic of crystalline selenium.

### EDS analysis

**Fig. 3:**
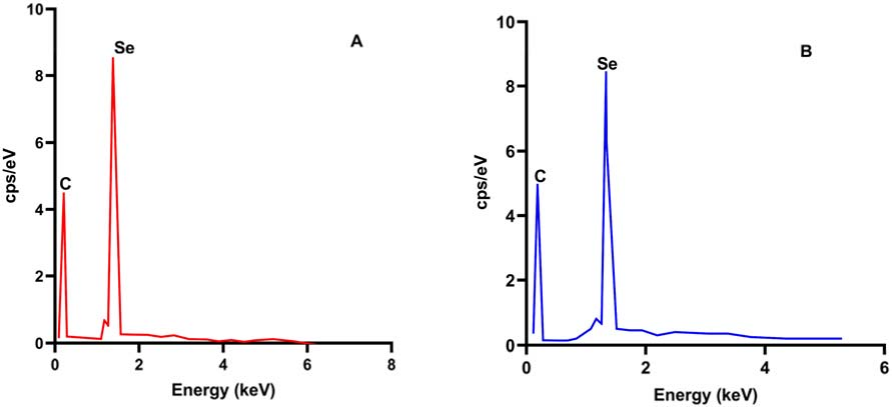
EDS spectrums of (A) red SeNPs and (B) grey SeNPs.

Following morphological characterization, energy-dispersive X-ray spectroscopy (EDS) was used to verify the elemental composition and assess the purity of both red and grey selenium nanoparticles Fig. 3.

EDS analysis confirmed selenium as the dominant elemental constituent in both red and grey SeNPs, with a characteristic Se Lα peak at ∼1.37 keV observed in all spectra. A carbon signal was also detected, which can be attributed to the carbon substrate used during SEM-EDS analysis. No additional elemental impurities were detected within the sensitivity limits of the technique, indicating comparable elemental purity for both allotropes. Importantly, EDS confirmed that the observed differences between red and grey SeNPs arise from structural and morphological variations rather than elemental composition.

### XRD analysis

**Fig. 4:**
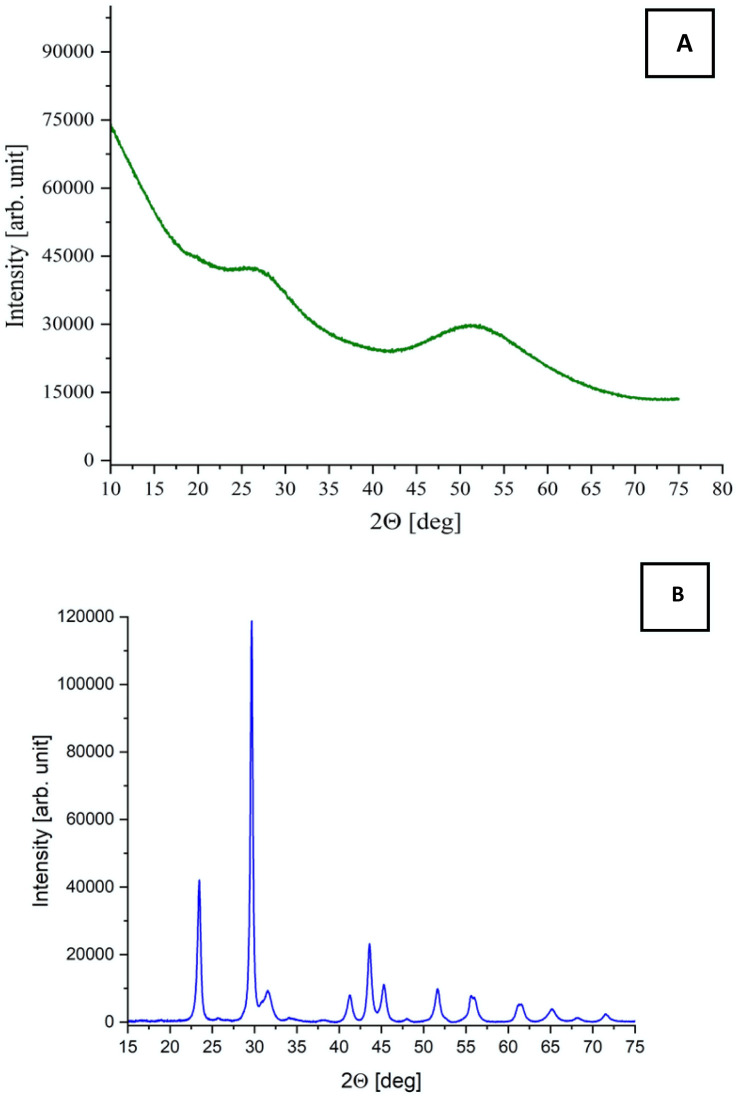
XRD patterns of (A) red SeNPs and (B) grey SeNPs.

To determine the structural nature and crystallinity of the synthesized selenium nanoparticles, X-ray diffraction (XRD) analysis was performed on both allotropes Fig. 4.

XRD analysis revealed a clear structural distinction between red and grey SeNPs. The diffraction pattern of red SeNPs contained only a broad diffuse halo centred between 20 and 30° 2*θ*, with no sharp Bragg reflections, indicating an amorphous structure lacking long-range atomic order. The broad feature is characteristic of disordered selenium containing only short-range Se–Se correlations. In contrast, grey SeNPs exhibited several sharp diffraction peaks at approximately 23.5°, 29.7°, 41.3°, 43.7°, 45.4°, 51.8°, 55.9°, and 61.5° 2*θ*, corresponding to the (100), (101), (110), (102), (111), (201), (202), and (210) planes of trigonal selenium according to ICDD PDF No. 06-0362. The sharper and narrower diffraction peaks of grey SeNPs indicate a higher degree of crystallinity and lower structural disorder than in red SeNPs. The reduced peak broadening suggests larger crystalline domains, lower lattice strain, and fewer structural defects.^29,30^

### Raman spectroscopy

**Fig. 5:**
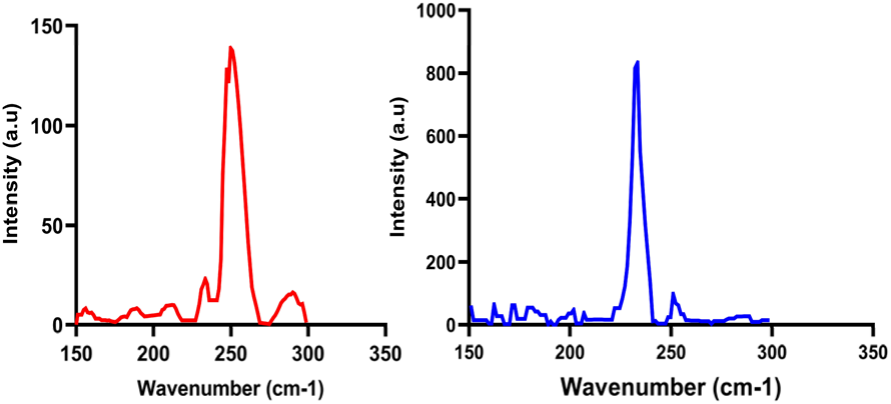
Raman spectra of selenium (redline) red Se. (blueline) grey Se.

Raman spectra of selenium are presented in Fig. 5; confirmed structural differences between red and grey SeNPs. The red SeNPs exhibited characteristic and weak peaks in the range of 250–255 cm^−1^, which are typically associated with the amorphous state of selenium, where structural disorder leads to peak broadening and poorly resolved vibrational modes. In contrast, the grey SeNPs showed distinct peaks between 230–235 cm^−1^, corresponding to the A_1_ vibrational mode of trigonal selenium associated with Se–Se stretching along helical chains. The narrow linewidth and high intensity of this peak indicate well-ordered atomic arrangements, in excellent agreement with the crystalline structure identified by XRD.

### Fluorescence analysis

**Fig. 6:**
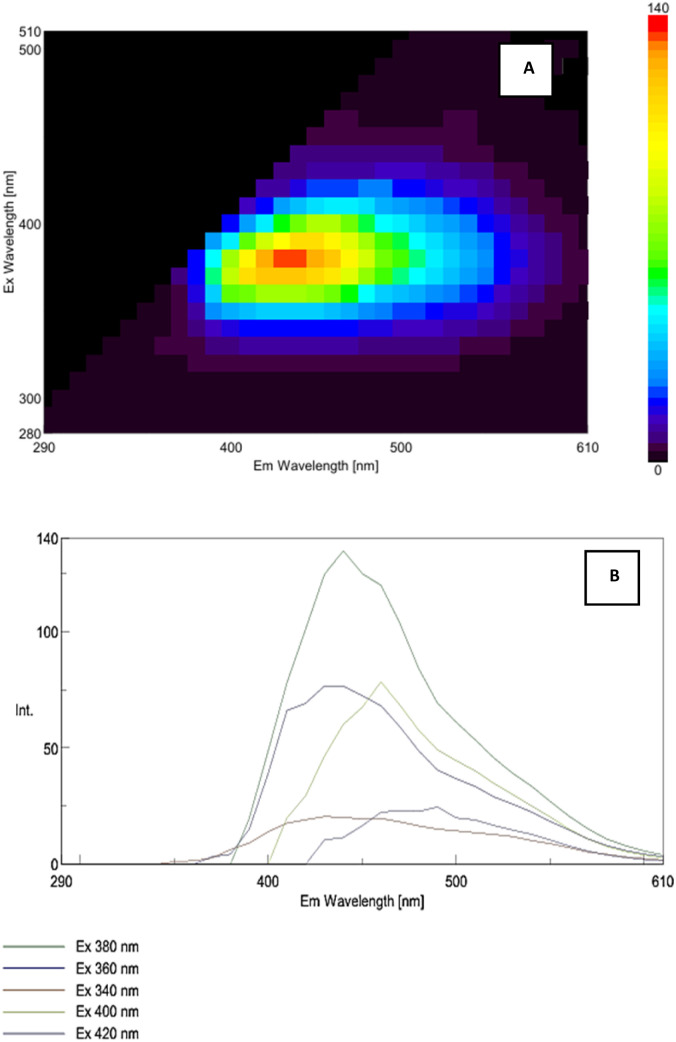
Fluorescence characteristics of red SeNPs at 1000 mg L^−1^: (A) 3D excitation–emission fluorescence spectrum of red SeNPs, and (B) 2D emission spectra recorded at different excitation wavelengths.

**Fig. 7:**
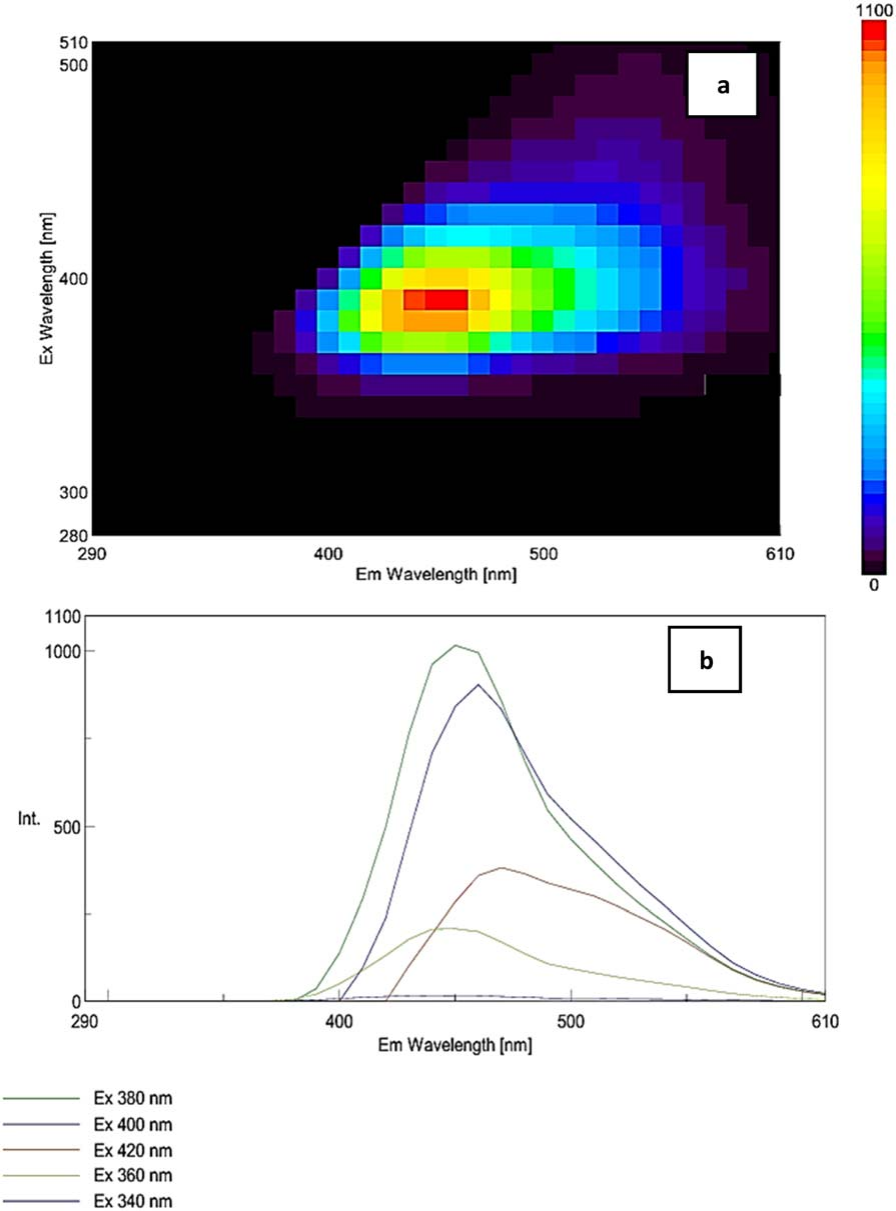
Fluorescence characteristics of grey SeNPs at 1000 mg L^−1^: (a) 3D excitation–emission fluorescence spectrum of grey SeNPs, and (b) 2D emission spectra recorded at different excitation wavelengths.

The optical properties of the amorphous red selenium nanoparticles were further investigated using excitation–emission fluorescence spectroscopy to evaluate their electronic and surface-related states Fig. 6 and 7.

The fluorescence characteristics of red SeNPs at 1000 mg L^−1^ were evaluated using full scan fluorescence analysis Fig. 6A. A weak fluorescence region was observed with the maximum emission around 430–450 nm when excited at ∼380 nm. The emission spectra recorded at selected excitation wavelengths Fig. 6B confirmed this behaviour, with the highest fluorescence intensity obtained at 380 nm excitation, followed by moderate signals at 360 nm and 400 nm, while 340 nm and 420 nm resulted in comparatively weaker emissions. The overall low intensity highlights the limited optical response of red SeNPs, distinguishing them from grey SeNPs, which displayed stronger fluorescence under similar conditions.

The fluorescence characteristics of grey SeNPs at 1000 mg L^−1^ were evaluated using full scan fluorescence analysis Fig. 7a. A well-defined fluorescence region was observed with the maximum emission centred around 430–450 nm when excited at ∼380–400 nm, indicating strong optical activity in the blue region. The emission spectra recorded at selected excitation wavelengths Fig. 7b confirmed this behaviour, with the highest fluorescence intensity obtained at 380 nm excitation, followed closely by 400 nm, while 420 nm, 360 nm, and 340 nm resulted in comparatively weaker emissions. The variation in intensity across ex-citation wavelengths highlights the excitation-dependent optical response of grey SeNPs. This behaviour is consistent with the strong optical activity of grey SeNPs, distinguishing them from red SeNPs, which exhibit lower fluorescence intensity under similar conditions.

### Fluorescence response of red and grey SeNPs

**Fig. 8:**
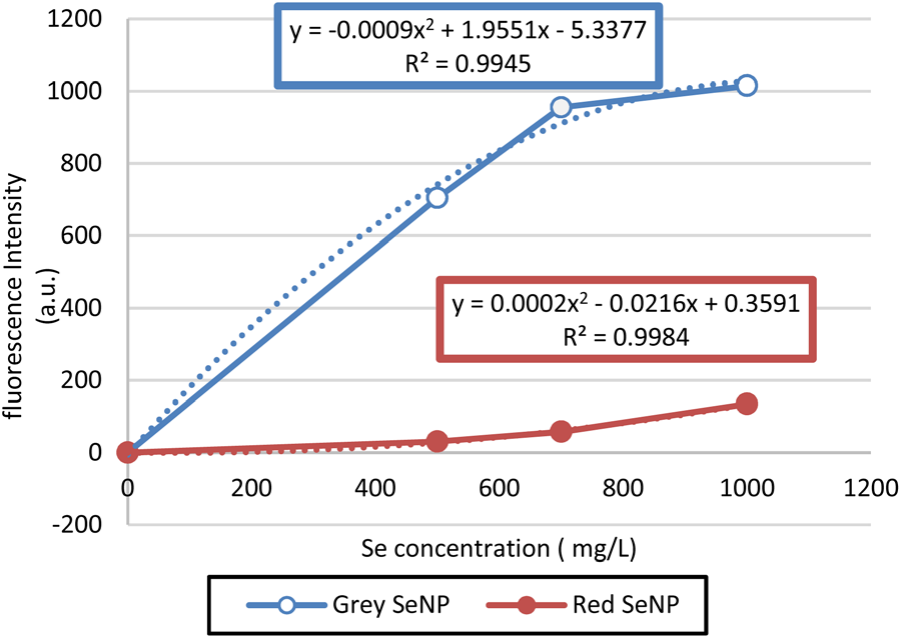
Fluorescence intensity of grey and red selenium nanoparticles at different concentrations (mg L^−1^).

Fluorescence measurements were performed on red and grey selenium nanoparticles (SeNPs). Fluorescence intensity was measured across a concentration range of Se (0–1000 mg L^−1^) in the sample solution (Fig. 8).

Grey SeNPs exhibited a concentration-dependent increase in fluorescence intensity, reaching a maximum near 1000 mg kg^−1^, with the relationship well described by a quadratic fit (*R*^2^ = 0.9945). In contrast, red SeNPs showed only a marginal increase in fluorescence intensity across the tested concentrations, with considerably lower emission compared to grey SeNPs (*R*^2^ = 0.9984). These findings highlight the stronger optical activity and concentration sensitivity of grey SeNPs relative to red SeNPs, suggesting distinct structural or surface-state contributions to their fluorescence behaviour.

## Discussion

The multitechnique characterization performed in this study demonstrates that red and grey selenium nanoparticles constitute two structurally and functionally distinct selenium allotropes at the nanoscale, despite having identical elemental composition. The combined SEM, XRD, Raman, and fluorescence analyses consistently show that the differences between the two SeNP systems arise from atomic ordering and crystallinity, which directly influence morphology and optical behavior.

The morphological differences observed by SEM clearly reflect the underlying structural organization of the two selenium allotropes. Red SeNPs exhibited predominantly spherical morphology with relatively uniform particle size, a feature commonly associated with amorphous selenium nanoparticles, where isotropic nucleation dominates due to the absence of long-range atomic order.^28,31,32^ Such morphology has been widely reported for red selenium synthesized under kinetically controlled conditions.

In contrast, grey SeNPs displayed elongated, needle-like nanostructures with high aspect ratios, which is characteristic of crystalline trigonal selenium. The anisotropic morphology observed here is consistent with directional growth along the helical Se–Se chains that define the trigonal selenium lattice.^17,28,33^ These SEM observations are fully supported by XRD analysis, which confirmed the amorphous nature of red SeNPs and the highly crystalline trigonal structure of grey SeNPs.^18,22,34–36^

Fluorescence and Raman spectroscopy were employed to characterize the optical and structural properties of the synthesized selenium nanoparticles (SeNPs). These complementary techniques provide insight into both the electronic transitions and vibrational modes of different selenium allotropes. Raman spectroscopy further confirmed the structural differences between the two selenium allotropes. Red SeNPs displayed broad peaks in the 250–255 cm^−1^ range, characteristic of the amorphous phase this broad feature arises from Se–Se stretching vibrations in disordered chain-like and Se8 ring-like structures, whereas grey SeNPs exhibited a sharp band at ∼233 cm^−1^, indicative of trigonal crystalline selenium and corresponding to the A_1_ stretching mode of ordered helical Se–Se chains in trigonal selenium.^16^ The partial transformation of amorphous selenium into crystalline form is consistent with earlier reports describing the coexistence and transition of selenium phases depending on synthesis conditions, stability, and external stress.^16^ According to previous studies, the amorphous phase typically shows Raman bands around 250 cm^−1^,^14,19^ while crystalline selenium occurs in two polymorphic forms: pure trigonal structure, characterized by the ∼233–237 cm^−1^ band observed in this study, and the monoclinic crystals, which appear near 251 cm^−1^,^24,25^ Raman spectroscopy is thus a powerful tool to distinguish these structural states, as selenium spectra are known to shift under oxidative and environmental conditions, reflecting its redox-sensitive nature.^23^ The optical properties of the SeNPs further support their nanoscale character. Biosynthesized SeNPs showed strong fluorescence activity in the 398–420 nm range, with excitation at 398 nm and emission intensities exceeding 800 a.u.^21^ Red SeNPs additionally exhibited an absorption band in the UV-vis spectrum at ∼260–270 nm, confirming their distinct optical activity.^27,37^ Such photoluminescence behaviour is strongly size- and surface-dependent, and selenium species are known to emit across the visible to near-infrared range.^26^ Notably, red SeNPs have been reported to fluoresce in the near-infrared region, making them suitable for biomedical applications such as imaging, diagnostics, and intracellular tracking.^38^ For comparison, selenite itself typically emits at ∼475 nm,^39^ highlighting the altered optical behaviour of nano selenium. However, the literature indicate that crystalline selenium nanostructures can indeed display significant fluorescence. The stronger fluorescence of grey SeNPs is likely related to their higher crystallinity and more ordered trigonal Se–Se chains, which reduce defect-related non-radiative recombination. In contrast, amorphous red SeNPs contain more structural defects and trap states that suppress fluorescence. Nevertheless, the observed optical differences cannot be attributed exclusively to allotropy, since crystallinity, morphology, and particle size also vary simultaneously. Therefore, the stronger fluorescence of grey SeNPs is most reasonably interpreted as arising from the combined effects of allotropy, crystallinity, and morphology, with structural ordering being the dominant factor. Surface states, defect density, and morphology-dependent scattering may also contribute to the observed optical behaviour. For example, For example, B. Gates and co-workers observed optical activity in trigonal selenium nanowires,^40^ while a more recent study demonstrated that selenium quantum dots with a trigonal structure exhibit strong solid-state fluorescence, with emission peaks varying between 418 and 449 nm depending on excitation wavelength.^24^ These findings demonstrate that red and grey SeNPs represent two structurally and electronically distinct selenium allotropes at the nanoscale. Red SeNPs are characterized by amorphous structure, spherical morphology, heterogeneous vibrational and electronic states, and weak fluorescence emission, whereas grey SeNPs exhibit high crystallinity, anisotropic needle-like morphology, well-defined vibrational modes, and strong fluorescence response. Baseline correction and background subtraction were applied during fluorescence analysis to minimize scattering and inner-filter effects. Although morphology-dependent scattering and surface chemistry may contribute partially to the enhanced emission of grey SeNPs, the results suggest that increased crystallinity remains the dominant factor. Similar relationships between allotropy, crystallinity, and optical behavior have also been reported in other chalcogen nanomaterials, including sulfur and tellurium, where structural phase and morphology strongly influence electronic and photophysical properties.^41,42^

## Conclusions

The present study demonstrates clear allotrope-dependent differences between red and grey selenium nanoparticles, despite their identical elemental composition. Red SeNPs possess an amorphous structure with spherical morphology, whereas grey SeNPs exhibit a crystalline trigonal structure and needle-like morphology, as confirmed by SEM, XRD, Raman, and fluorescence analyses. Optical characterization revealed that grey SeNPs exhibit stronger and more defined fluorescence emission than red SeNPs, indicating that crystallinity and structural ordering enhance the photophysical properties of selenium nanoparticles. In contrast, the amorphous red SeNPs displayed weaker, excitation-dependent fluorescence, reflecting greater structural disorder. Taken together, these findings show that selenium allotropy significantly influences the structural and optical properties of selenium nanoparticles and provides a physicochemical basis for interpreting their different biological behaviors their promise for applications in optical imaging, biosensing, and diagnostic technologies.

## Author contributions

Conceptualization and methodology were carried out by A. F.; the experiments were conducted by A. F.; A. F. performed the data analysis and prepared the original draft.; investigation, A. F. and A. C.; supervision, J. P.; A. F. and J. P contributed to the review and editing of the manuscript. All authors have read and agreed to the published version of the manuscript.

## Conflicts of interest

There are no conflicts to declare.

## Data Availability

The data supporting this article, including all experimental data and analyses discussed, are available within the article.
